# Mycothiol synthesis by an anomerization reaction through endocyclic cleavage

**DOI:** 10.3762/bjoc.12.35

**Published:** 2016-02-22

**Authors:** Shino Manabe, Yukishige Ito

**Affiliations:** 1 Synthetic Cellular Chemistry Lab, RIKEN, Hirosawa, Wako, Saitama 351-0198, Japan

**Keywords:** anomerization, aminoglycoside, endocyclic cleavage reaction, inositol, mycothiol

## Abstract

Mycothiol is found in Gram-positive bacteria, where it helps in maintaining a reducing intracellular environment and it plays an important role in protecting the cell from toxic chemicals. The inhibition of the mycothiol biosynthesis is considered as a treatment for tuberculosis. Mycothiol contains an α-aminoglycoside, which is difficult to prepare stereoselectively by a conventional glycosylation reaction. In this study, mycothiol was synthesized by an anomerization reaction from an easily prepared β-aminoglycoside through endocyclic cleavage.

## Introduction

Tuberculosis is an infectious disease and has had a high death rate over the past few decades [1–4]. The occurrence of multiple-drug-resistant (MDR), extensive-drug-resistant (EDR), and totally drug-resistant (TDR) pathogens has increased the need for new drug candidates for treating tuberculosis.

**Figure 1:**
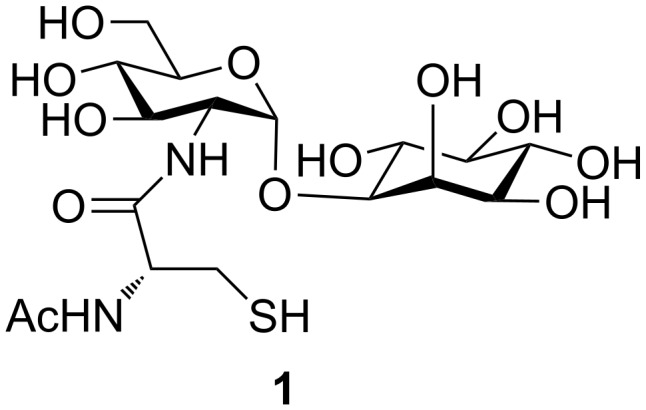
Structure of mycothiol **1**.

Mycothiol (MSH) **1** is the main low-molecular-weight thiol found in most actinomycetes, including *Mycobacteria* and *Streptomycetes* [5–10]. It consists of an *N*-acetylcysteine, a D-glucosamine, and a D-*myo*-inositol moiety (Figure 1). D-Glucosamine is α-linked to D-*myo*-inositol at the 1-position, and *N*-acetylcysteine is linked to the amino group of D-glucosamine. The conformation of MSH has been investigated by NMR analyses and computational calculations [11–12]. Recently, *N*-acyl variants of MSH homologs, such as formyl, propanoyl, and succinoyl, have been reported [13–15].

**Scheme 1:**
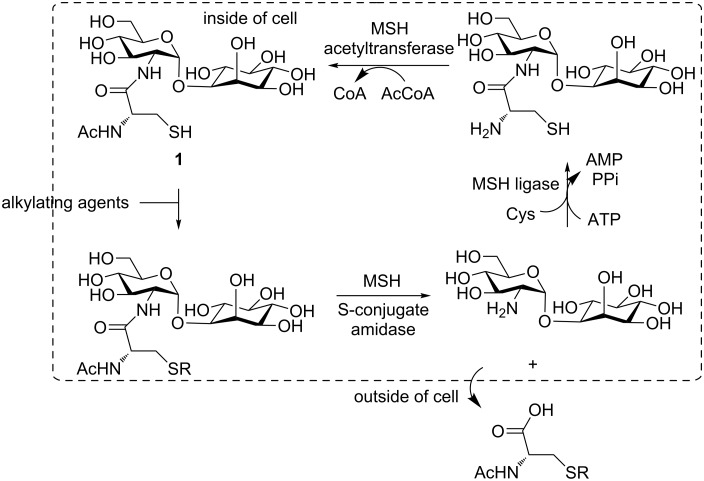
Detoxification pathway mediated by MSH.

Gram-negative bacteria and most Eukaryotes utilize glutathione as a low-molecular-weight thiol for maintaining a reducing environment in the cytosol. Gram-positive bacteria including actinomycetes lack glutathione, instead, MSH is found as the major low-molecular-weight thiol. It is considered that MSH is required for maintaining a reducing intracellular environment in Gram-positive bacteria, similar to glutathione in eukaryotes and Gram-negative bacteria. MSH undergoes metal-catalyzed autoxidation more rapidly than glutathione [16]. The biosynthetic pathway of MSH has been well investigated; MSH is synthesized from 1-inositol phosphate and uridine diphosphate *N*-acetylglucosamine (UDP-GlcNAc) in five steps [15]. It is used by mycobacteria for protection against foreign electrophilic agents such as oxidants, radicals, and drugs. In the detoxification pathway, MSH reacts with alkylating reagents and the resulting *S*-conjugates are subsequently cleaved at the amide bond by MSH *S*-conjugate amidase (Scheme 1) [4–10]. After cleavage, the *N-*acetylcysteine *S*-conjugate is transported out of the cell, while the inositol–glucosamine conjugate is recycled to afford MSH. MSH also plays an important role in the growth and survival of *Mycobacterium tuberculosis*. Because MSH-dependent pathways are not found in eukaryotes, the enzymes involved may be considered as novel antimicrobacterial targets, especially for tuberculosis, and several compounds with inhibitory activity have been synthesized [17–18]. In addition, another function of MSH was recently reported; it is involved in the biosynthesis of lincomycin A, a sulfur-containing lincosamide antibiotic [19].

Due to the limited availability of MSH from *M. smegmatis* cell culture (<1.5 mg of MSH from 1 L culture) [20], the chemical synthesis of MSH is highly desired. Bewley et al. and Lee and Rosazza independently reported the synthesis of MSH and determined the absolute stereochemistry of the glucosamine and cysteine moieties [21–22]. Hung also reported the utility of the 2-azido-2-deoxyglycosyl donor and resolved inositol isomers in a recent MSH synthesis [23]. Knapp et al. reported an intramolecular aglycone delivery method in order to achieve complete α-stereoselectivity [24]. The Ni(4-F-PhCN)_4_(OTf)_2_-catalyzed synthesis of *N*-substituted benzylideneaminoglycosides has also been achieved to fabricate MSH [25]. The α-stereoselective formation of aminoglycoside is the crucial step in MSH synthesis. However, except for the intramolecular aglycone delivery method developed by Knapp et al., complete α-stereoselective glycosylation reactions were difficult in mycothiol synthesis. The complete α-stereoselective glycosylation reaction of aminoglycosides is still generally difficult at this moment [26–28].

**Scheme 2:**
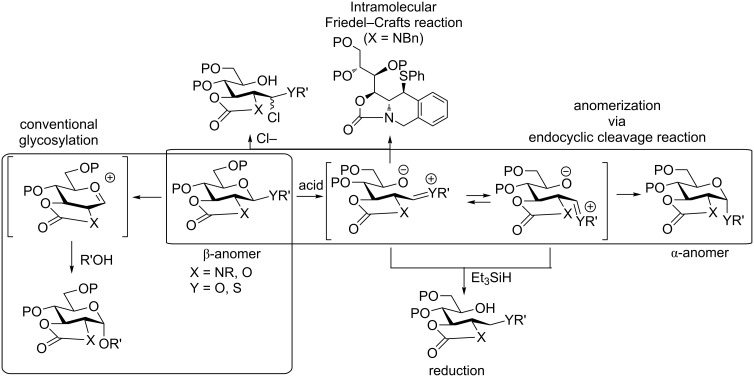
Anomerization via endocyclic cleavage.

Oscarson and our group recently demonstrated that reactions of pyranosides with *N*-acetyl 2,3-*trans*-carbamate groups exhibited complete anomerization from β-glycoside to α-glycoside in the presence of a weak Lewis acid through an endocyclic cleavage reaction [29–33]. We showed evidence of the endocyclic cleavage reaction by trapping linear cations through reduction, and intramolecular Friedel–Crafts reaction [29–30] (Scheme 2). In particular, the reaction of pyranosides bearing acetyl substituents on the carbamate groups showed complete anomerization [32]. We expected that anomerization via endocyclic cleavage would be useful for mycothiol synthesis.

## Results and Discussion

**Scheme 3:**
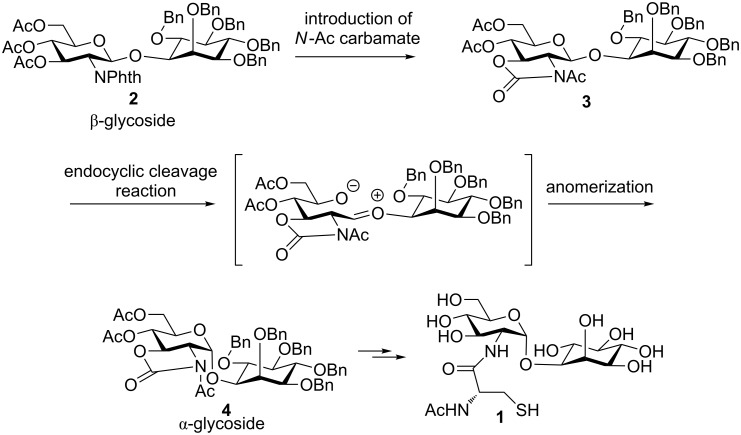
Outline of mycothiol synthesis by anomerization.

Based on the results of our previous study, we expected that an anomerization would be useful for the stereoselective synthesis of α-aminoglycosides, which is normally difficult by conventional glycosylation reactions. β-Glycoside **2**, which is synthesized by assistance from the phthalimide group in the 2-position, was converted to α-glycoside **4**, by introducing an *N*-acetyl 2,3-*trans*-carbamate group (Scheme 3) and by conducting an anomerization reaction.

**Scheme 4:**
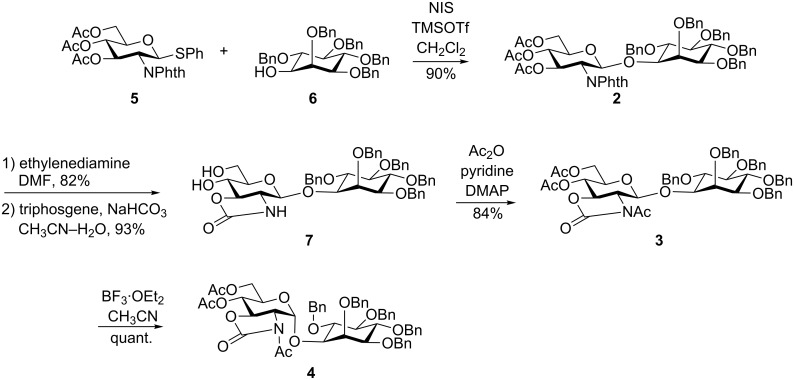
Synthesis of a pseudodisaccharide by an anomerization reaction.

The glycosylation reaction of phthalimido-protected glucosamine thioglycoside **5** with inositol **6** [24] afforded β-linked pseudo-disaccharide **2** in 90% yield (Scheme 4). After removing the phthaloyl and acetyl groups by using ethylenediamine in dimethylformamide (DMF), a carbamate group was introduced using triphosgene in the presence of NaHCO_3_. Acetylation of both the hydroxy and carbamate groups was carried out using acetic anhydride in pyridine in the presence of 4-dimethylaminopyridine (DMAP). Finally, β-glycoside **3** was completely anomerized to the corresponding α-anomer **4** in quantitative yield in the presence of two equivalents of BF_3_·OEt_2_ in CH_3_CN [33] within 30 min at −30 °C.

**Scheme 5:**
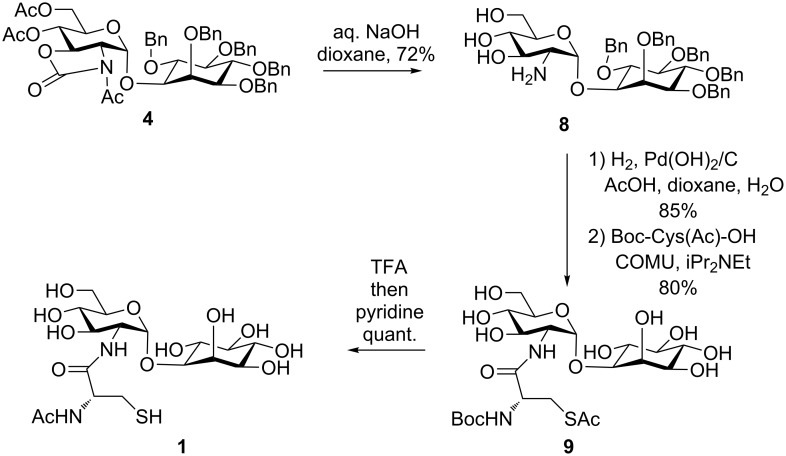
Mycothiol synthesis from pseudo-disaccharide **4**.

With the α-linked inositol **4** in hand, the synthesis of MSH was completed as follows (Scheme 5): the carbamate and acetyl groups were removed by alkaline hydrolysis to give known compound **8** [24]; then, benzyl groups were removed by H_2_/Pd(OH)_2_/C in AcOH/dioxane/H_2_O. Although it was reported that the cysteine moiety was introduced by 1-[bis(dimethylamino)methylene]-1*H*-1,2,3-triazolo[4,5-*b*]pyridinium 3-oxid hexafluorophosphate (HATU) [23–24], we found that purification of the product was rather difficult, especially for >50 mg-scale reactions. The reason for the low yield is probably the low solubility of the product in the reported solvent system during column chromatography (CHCl_3_/MeOH/AcOH), and azabenzotriazole from HATU was difficult to remove. Instead, ethyl (hydroxyimino)cyanoacetate (COMU) [34] coupling gave product **9** in 80% yield after reversed-phase column chromatography purification (H_2_O/MeOH). Finally, Boc removal and subsequent acetyl migration, as reported previously, gave mycothiol **1** [23–24].

## Conclusion

We demonstrated a MSH synthesis using an anomerization reaction through endocyclic cleavage in the presence of a weak Lewis acid. Murphy also reported the utility of the anomerization of glucuronic acid for preparing 1,2-*cis*-linked glycolipids [35–37]. Sulfated sugars are isomerized from pyranosides to furanosides [38]. The anomerization reaction would be a useful methodology to prepare 1,2-*cis*-glycosides such as heparin and glycosylphosphatidylinositol (GPI) anchors.

## Supporting Information

File 1 Experimental procedures, spectral data of new compounds, including ^1^H and ^13^C NMR spectra.
