# Supplementary material for: Mycothiol synthesis by an anomerization reaction through endocyclic cleavage
Source: Beilstein J Org Chem. 2016 Feb 22;12:328–33. doi: 10.3762/bjoc.12.35 (PMC4778527; doi:10.3762/bjoc.12.35)

Supporting Information
for

Mycothiol synthesis by an anomerization reaction through endocyclic cleavage

Shino Manabe* and Yukishige Ito*
Address: Synthetic Cellular Chemistry Lab, RIKEN, Hirosawa, Wako, Saitama 351-0198, Japan
Email: Shino Manabe - smanabe@riken.jp; Yukishige Ito - yukito@riken.jp
*Corresponding author

Experimental procedures, spectral data of new compounds, including ^{1}H and ^{13}C NMR spectra.

Contents

General methods and experimental procedures ············ S2
^{1}H NMR spectrum of **2** ············ S6
^{13}C NMR spectrum of **2** ············ S7
^{1}H NMR spectrum of intermediate aminoalcohol ············ S8
^{13}C NMR spectrum of intermediate aminoalcohol ············ S9
^{1}H NMR spectrum of **7** ············ S10
^{13}C NMR spectrum of **7** ············ S11
^{1}H NMR spectrum of **3** ············ S12
^{13}C NMR spectrum of **3** ············ S13
^{1}H NMR spectrum of **4** ············ S14
^{13}C NMR spectrum of **4** ············ S15
^{1}H NMR spectrum of **8** ············ S16
^{13}C NMR spectrum of **8** ············ S17
^{1}H NMR spectrum of debenzylated **8** ············ S18
^{13}C NMR spectrum of debenzylated **8** ············ S19
^{1}H NMR spectrum of **9** ············ S20
^{13}C NMR spectrum of **9** ············ S21
^{1}H NMR spectrum of mycothiol **1** ············ S22
^{13}C NMR spectrum of mycothiol **1** ············ S23

General methods and experimental procedures:

All commercial reagents were used without further purification. Analytical TLC was performed on silica gel 60 F254 plates (Merck) and visualized by UV fluorescence quenching and 12 Molybdo(VI) phosphoric acid acid/phosphoric acid /sulfuric acid staining. Flash column chromatography was performed on silica gel 60N (spherical, neutral, 40–100 μm, Kanto Co.). Yields reported here are isolated yields. ^{1}H and ^{13}C NMR spectra were recorded on a JEOL EX 400 spectrometer (400 and 100 MHz, respectively) at ambient temperatures (23–24 °C) in $CDCl_3$, CD_3OD and D_2O. Chemical shifts (δ) are reported in ppm relative to internal TMS (δ = 0.00 ppm) in $CDCl_3$, or remaining solvent peak (δ = 3.30 ppm for CD_3OD) and (δ = 3.34 ppm for D_2O) for ^{1}H NMR spectra. $CDCl_3$ (δ = 77.00 ppm), CD_3OD (δ = 49.00 ppm) for ^{13}C NMR spectra. For ^{13}C spectra in D_2O, MeOH (δ = 49.50 ppm as CH_3) was used as an internal standard. HRMS were measured by quadrupole–TOF mass spectrometry. Optical rotations were measured at room temperature (JASCO DIP-310).

Compound 2: To a solution of acceptor **6** (230 mg, 0.365 mmol) and donor **5** (250 mg, 0.475 mmol) in CH_2Cl_2 (3 mL), NIS (106 mg, 0.475 mmol) and TMSOTf (9 μL, 0.048 mmol) were added at – 30 °C. After 5 h, the reaction was quenched with $Na_2S_2O_3$. The aqueous layer was extracted with EtOAc. The combined layers were washed with sat. $NaHCO_3$ and brine. After drying the extract over Na_2SO_4, the solvent was removed under reduced pressure. The residue was purified by size-exclusion column chromatography (Bio-Beads X-3, toluene) and silica gel column chromatography (hexane/EtOAc 7:3–1:1) to give 344 mg of product **2** (90%). $[\alpha]^{24}_{D}$ = 5.10 (*c* = 0.99, $CHCl_3$); ^{1}H NMR (400 MHz, $CDCl_3$, TMS): δ 7.46–7.08 (m, 26H), 6.91 (d, *J* = 7.6 Hz, 2H), 6.87 (d, *J* = 7.6 Hz), 5.80 (t, *J* = 10.8 Hz, 1H), 5.61 (d, *J* = 8.4 Hz, 1H), 5.12 (t, *J* = 10.0 Hz, 1H), 4.88–4.67 (m, 7H), 4.51 (d, *J* = 10.0 Hz, 1H), 4.44–4.37 (m, 2H), 4.27–4.13 (m, 4H), 3.98 (t, *J* = 9.6 Hz, 1H), 3.86–3.81 (m, 2H), 3.57 (d, *J* = 10.0, 2.4 Hz, 1H), 3.39–3.33 (m, 2H), 2.04 (s, 3H), 1.99 (s, 3H), 1.83 (s, 3H); ^{13}C NMR (100 MHz, $CDCl_3$): δ 170.4, 170.0, 169.5, 139.2, 138.8, 138.6, 138.4, 133.9, 128.3, 128.3, 128.1, 128.1, 128.0, 127.9, 127.7, 127.5, 127.3, 127.3, 126.6, 126.0, 123.4, 99.1, 83.6, 81.4, 81.3, 80.6, 80.4, 75.7, 75.7, 74.7, 74.5, 72.5, 71.7, 70.5, 69.9, 61.9, 55.0, 20.7, 20.6, 20.4; HRMS: *m/z* calcd for $C_{61}H_{61}NO_{15}$ + Na^+: 1070.3933 [*M* + Na^+]; found 1070.3933.

Compound 7: A solution of phthalimide **2** (138 mg, 0.131 mmol) and ethylenediamine (0.2 mL) in DMF (1 mL) was stirred at 80 °C under Ar atmosphere overnight. After cooling the mixture to room temperature, the mixture was concentrated under vacuum, and the residue was purified by LH-20 ($CHCl_3$/MeOH, 1:1) and silica gel column

chromatography to afford aminoalcohol (85.6 mg, 82% yield). [α] = −17.9 (c = 1.0, MeOH); ^{1}H NMR (400 MHz, CD_3OD): δ 7.44 (d, J = 7.2 Hz, 2H), 7.33–7.21 (m, 23H), 4.94–4.73 (m, 13H), 4.67 (d, J = 11.6 Hz, 1H), 4.58 (d, J = 11.6 Hz, 1H), 4.48 (s, 1H), 4.43 (d, J = 7.6 Hz, 1H), 3.97 (t, J = 9.6 Hz, 1H), 3.92 (dd, J = 11.6, 2.8 Hz, 1H), 3.70 (dd, J = 9.6 Hz, 2.8 Hz, 1H), 3.65 (dd, J = 12.0 Hz, 6.4 Hz, 1H), 3.54 (dd, J = 12.0 Hz, 2.8 Hz, 1H), 3.49 (t, J = 8.8 Hz, 1H), 3.31–3.20 (m, 4H), 2.61 (t, J = 8.8 Hz, 1H); ^{13}C NMR (100 MHz, CD_3OD): δ 140.9, 140.2, 140.1, 140.0, 129.3, 129.2, 129.1, 129.0, 128.9, 128.7, 128.7, 128.6, 128.5, 128.5, 128.3, 106.4, 85.0, 82.8, 82.3, 82.3, 81.7, 78.5, 78.2, 77.8, 76.7, 76.5, 76.4, 75.9, 73.2, 72.1, 63.2, 59.2; HRMS: m/z calcd for $C_{47}H_{53}NO_{10}$ + Na^+ 814.3562, [M + Na^+]; found 814.3561.

To a solution of aminoalcohol (50 mg, 0.063 mmol) in CH_3CN (1 mL) and H_2O (0.5 mL), $NaHCO_3$ (51 mg, 0.61 mmol) and triphosgene (18 mg, 0.061 mmol) were added. After overnight stirring, the reaction mixture was diluted with $CHCl_3$ and brine. After the separation of layers, the aqueous layer was extracted with $CHCl_3$. The combined layers were washed with brine and dried over Na_2SO_4. After concentration under reduced pressure, the residue was purified by silica gel column chromatography to afford the product **7** (48.0 mg, 93% yield). $[\alpha]^{24}_D$ = 83.9 (c = 0.84, $CHCl_3$); ^{1}H NMR (400 MHz, $CDCl_3$, TMS): δ 7.40–7.27 (m, 25H), 4.96–4.89 (m, 4H), 4.84 (d, J = 10.4 Hz, 1H), 4.83 (d, J = 10.8 Hz, 1H), 4.77 (d, J = 7.6 Hz, 1H), 4.74 (s, 1H), 4.71 (s, 2H), 4.64 (d, J = 11.6 Hz, 1H), 4.63 (d, J = 10.8 Hz, 1H), 4.11–4.93 (m, 4H), 3.86 (t, J = 11.2 Hz, 1H), 3.76–3.83 (m, 2H), 3.50–3.43 (m, 2H), 3.40 (dd, J = 9.6, 2.4 Hz, 1H); ^{13}C NMR (100 MHz, $CDCl_3$); δ 158.7, 139.1, 138.6, 138.4, 138.2, 138.2, 128.6, 128.4, 128.4, 128.2, 128.1, 128.0, 127.8, 127.7, 127.6, 127.5, 101.9, 83.8, 81.7, 81.6, 81.3, 80.5, 79.3, 77.8, 77.7, 75.9, 74.6, 73.1, 67.8, 61.5, 59.6; HRMS: m/z calcd for $C_{48}H_{51}NO_{11}$ + Na^+: 840.3354, [M + Na^+]; found 840.3349.

Compound 3: A mixture of carbamate **7** (50 mg, 0.0612 mmol) and DMAP (3 mg, 0.0246 mmol) in Ac_2O (1 mL) and pyridine (1 mL) was stirred overnight. After evaporation, the residue was purified by silica gel column chromatography (hexane/EtOAc 7:3) to afford **3** (48.2 mg, 84% yield). ^{1}H NMR (400 MHz, $CDCl_3$, TMS): δ 7.54–7.52 (m, 2H), 7.36–7.17 (m, 23H), 5.20 (t, J = 10.0 Hz, 1H), 5.18 (d, J = 8.8 Hz, 1H), 5.01 (d, J = 11.6 Hz, 1H), 4.94–4.60 (m, 4H), 4.70–4.31 (m, 4H), 4.12 (m. 1H), 4.12–3.94 (m, 6H), 3.90 (m, 1H), 3.74 (m, 1H), 3.62–3.42 (m, 2H), 2.52 (s, 3H), 2.28 (s, 3H), 1.98 (s, 3H); ^{13}C NMR (100 MHz, $CDCl_3$): δ 170.7, 170.3, 169.2, 153.4, 139.1, 138.9, 138.7, 138.6, 138.4, 128.4, 128.3, 128.3, 128.2, 128.1, 128.1, 127.9, 127.6, 127.5, 127.4, 127.3, 126.8, 102.2, 84.0, 81.6, 81.4, 80.5, 78.7, 76.6, 76.2, 75.8, 75.6, 75.1, 75.0, 74.2, 72.2, 68.1, 62.2, 60.4, 24.6, 20.6; HRMS: m/z calcd for $C_{54}H_{57}NO_{14}$ + Na^+: 966.3671 [M + Na^+]; found: 966.3668.

Compound 4: To a solution of β-glycoside **3** (50.0 mg, 0.530 mmol) in CH_3CN (0.69 mL), $BF_3 \cdot OEt_2$ (14 μL, 0.106 mmol) was added at −30 °C. After 20 min, the reaction was quenched with saturated $NaHCO_3$, and the aqueous layer was extracted with EtOAc. The combined layer was washed with brine and dried over Na_2SO_4. After concentration, the residue was purified by silica gel column chromatography (hexane/EtOAc, 7:3–1:1) to afford α-glycoside **4** (50.0 mg, quant.). [α] = 49.6 (*c* = 1.42, $CHCl_3$); ^1H NMR (400 MHz, $CDCl_3$, TMS): δ 7.37–7.23 (m, 25H), 5.83 (s, 1H), 5.19 (t, *J* = 9.6 Hz, 1H), 5.08–5.03 (m, 2H), 4.83–4.71 (m, 5H), 4.54–4.44 (m, 2H), 4.11–4.07 (m, 4H), 3.99 (d, *J* = 12.0 Hz, 1H), 3.85–3.77 (m, 2H), 3.65 (d, *J* = 10.4 Hz, 1H), 3.54 (t, *J* = 8.8 Hz, 1H), 3.47 (d, *J* = 9.6 Hz, 1H), 2.06 (s, 3H), 2.00 (s, 3H), 1.96 (s, 3H); ^{13}C NMR (100 MHz, $CDCl_3$): δ 171.7, 170.5, 169.0, 152.4, 139.4, 138.6, 138.4, 138.3, 138.0, 128.5, 128.4, 128.3, 128.2, 128.0, 127.8, 127.7, 127.6, 127.5, 127.2, 126.2, 93.0, 84.2, 81.8, 81.2, 79.1, 76.0, 76.0, 75.9, 75.1, 74.8, 74.6, 74.0, 72.9, 69.6, 67.7, 60.9, 59.8, 23.2, 20.7, 20.5; HRMS: *m/z* calcd for $C_{54}H_{57}NO_{14} + Na^+$: 966.3671 [$M + Na^+$]; found: 966.3668.

Compound 8:[1] A solution of carbamate **4** (150 mg, 0.159 mmol) in 10% NaOH and dioxane was heated at 100 °C for 24 h. After the mixture was neutralized with 2 M HCl and concentration, the residue was purified by silica gel column chromatography ($CHCl_3$:MeOH 98:2-9:1) to give compound **8** (95.4 mg, 72 %). ^1H NMR (400 MHz, CD_3OD) δ 7.45-7.21 (25H), 4.94-4.65 (m, 10H), 4.67 (d, *J* = 11.6 Hz, 1H), 4.58 (d, *J* = 11.6 Hz, 1H), 4.48 (s, 1H), 4.43 (d, *J* = 7.6 Hz, 1H), 3.99-3.94 (m, 3H), 3.72-3.66 (m, 2H), 3.55 (d, *J* = 12.4 Hz, 1H), 3.49 (t, 3H), 3.31-3.18 (m, 4H), 2.61 (t, *J* = 8.4 Hz, 1H); ^{13}C NMR (100 MHz, CD_3OD): δ 140.6, 140.1, 140.1, 139.9, 139.8, 129.5, 129.4, 129.3, 129.2, 129.1, 128.8, 128.6, 128.5, 128.4, 96.6, 85.0, 82.8, 82.6, 81.4, 76.8, 76.6, 76.1, 75.9, 75.7, 75.0, 73.9, 73.7, 71.6, 62.4, 57.2; HRMS: m/z calcd for $C_{47}H_{53}NO_{10} + Na^+$: 814.3562 [$M + Na^+$]; found: 814.3561.

Compound 9:[1,2] A suspension of pentabenzyl compound (0.23 g, 0.291 mmol) and $Pd(OH)_2/C$ (100 mg) in dioxane (10 mL), H_2O (10 mL), and AcOH (5 mL) was stirred at room temperature for 1 day. The mixture was filtered and freezed-dried. The amine·AcOH salt was obtained (0.10 g, 85%).
The amine·AcOH salt was treated by DOWEX 1x8-100 ion exchange resin in order to remove AcOH. Then, to a solution of amine (50 mg, 0.150 mmol) and Boc-Cys(Ac)-OH (42 mg, 0.160 mmol) in DMF (1 mL), COMU (69 mg, 0.160 mmol) and iPr_2NEt (28 μL, 0.160 mmol) were added at 4 °C. After stirring the mixture at room temperature overnight, the mixture was purified by LH20 (H_2O/MeOH 1:1) and SepPak (H_2O/MeOH 9:1–4:1) to give compound **9** (70 mg, 80%).

Compound **1** was synthesized as previously reported [1, 2].

[1] Chung. C. C.; Zulueta, M. M. L.; Padiyar, L. T.; Hung, S.-C. *Org. Lett.* **2011**, *13*, 5496–5499.
[2] Ajayi, K.; Thakur, V. V.; Rapo, R. C.; Knapp, S. *Org. Lett*. **2010**, *12*, 2630–2633.

^{1}H NMR spectrum of **2**

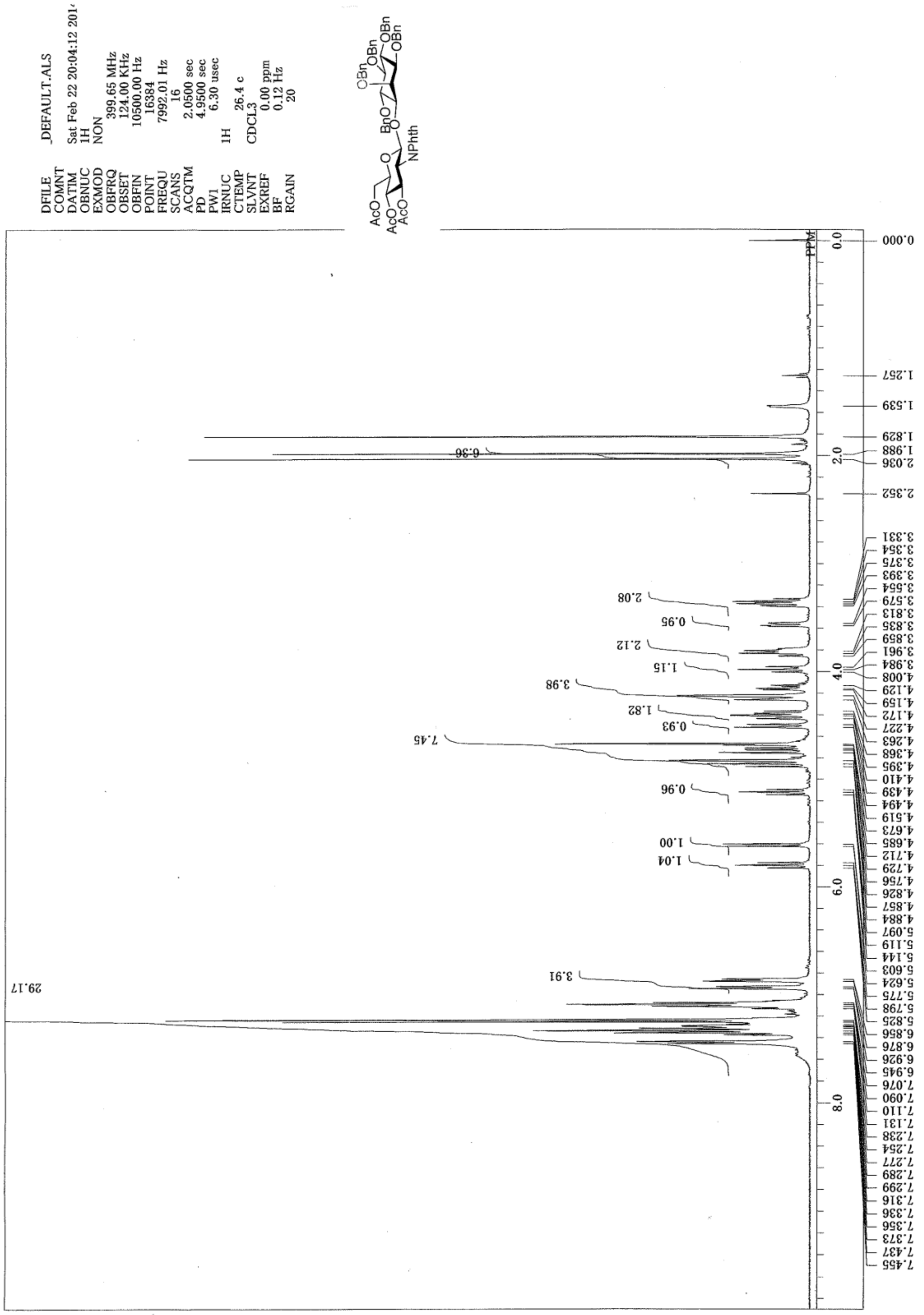

^{13}C NMR spectrum of **2**

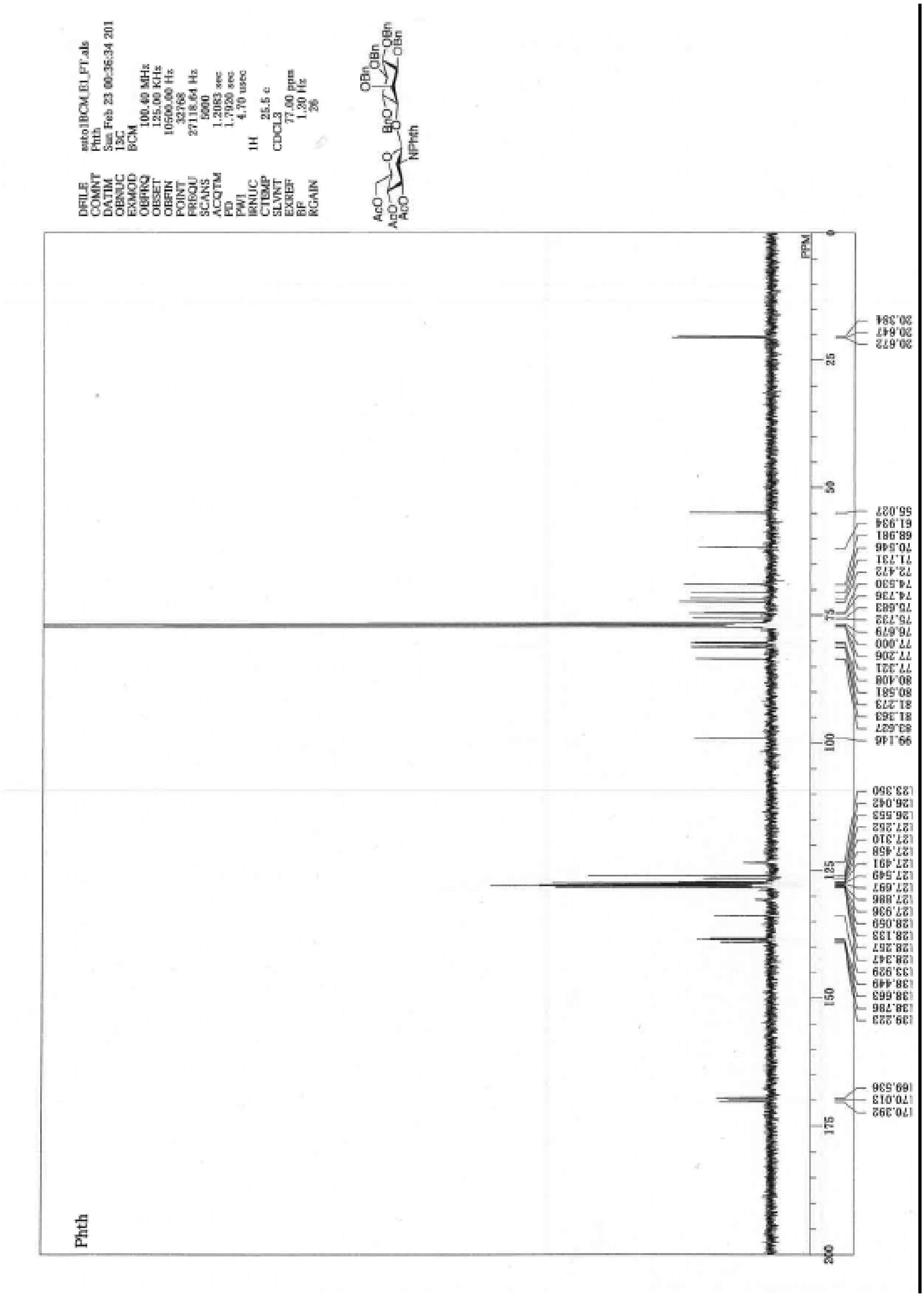

^{1}H NMR spectrum of intermediate aminoalcohol

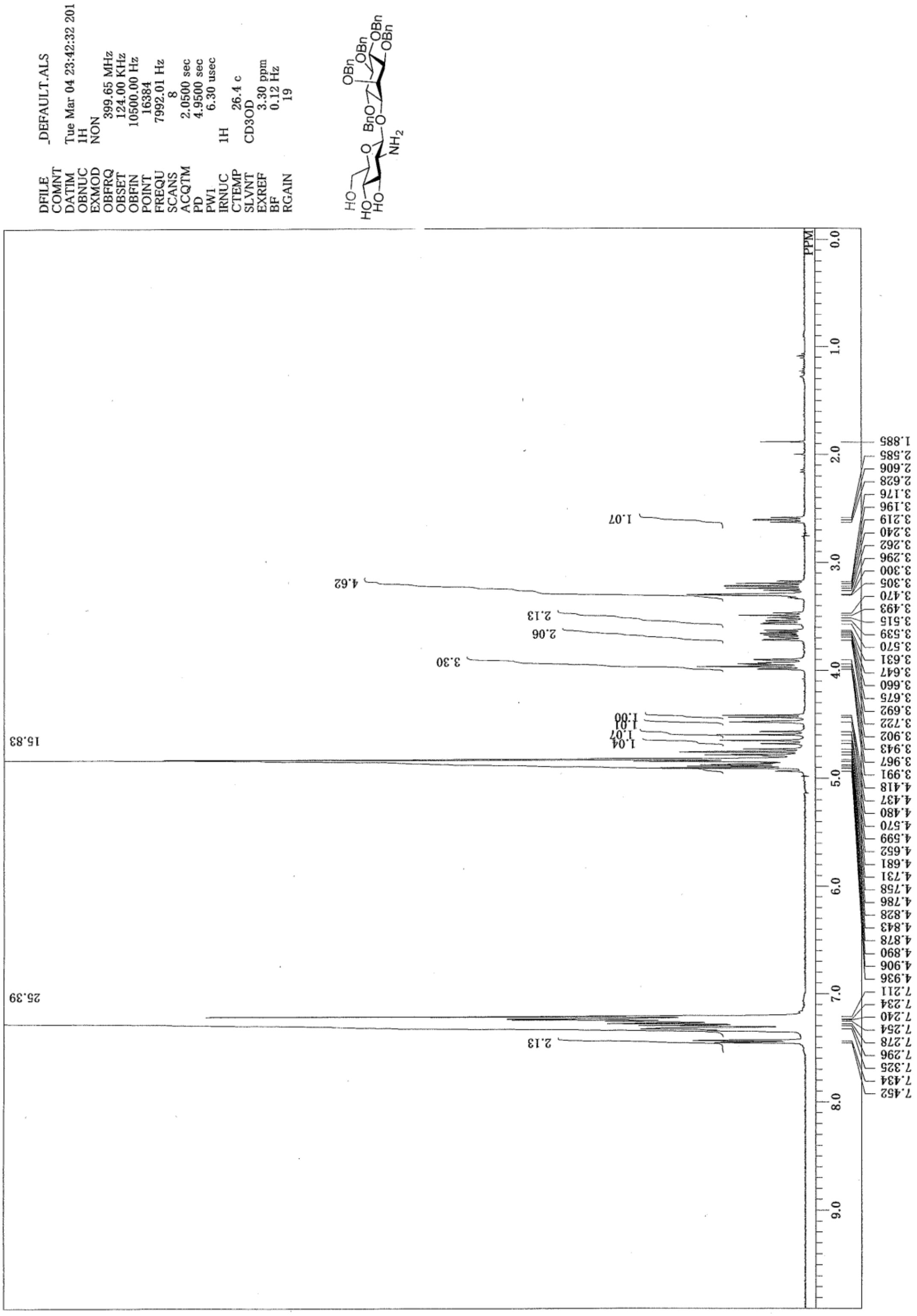

^{13}C NMR spectrum of intermediate aminoalcohol

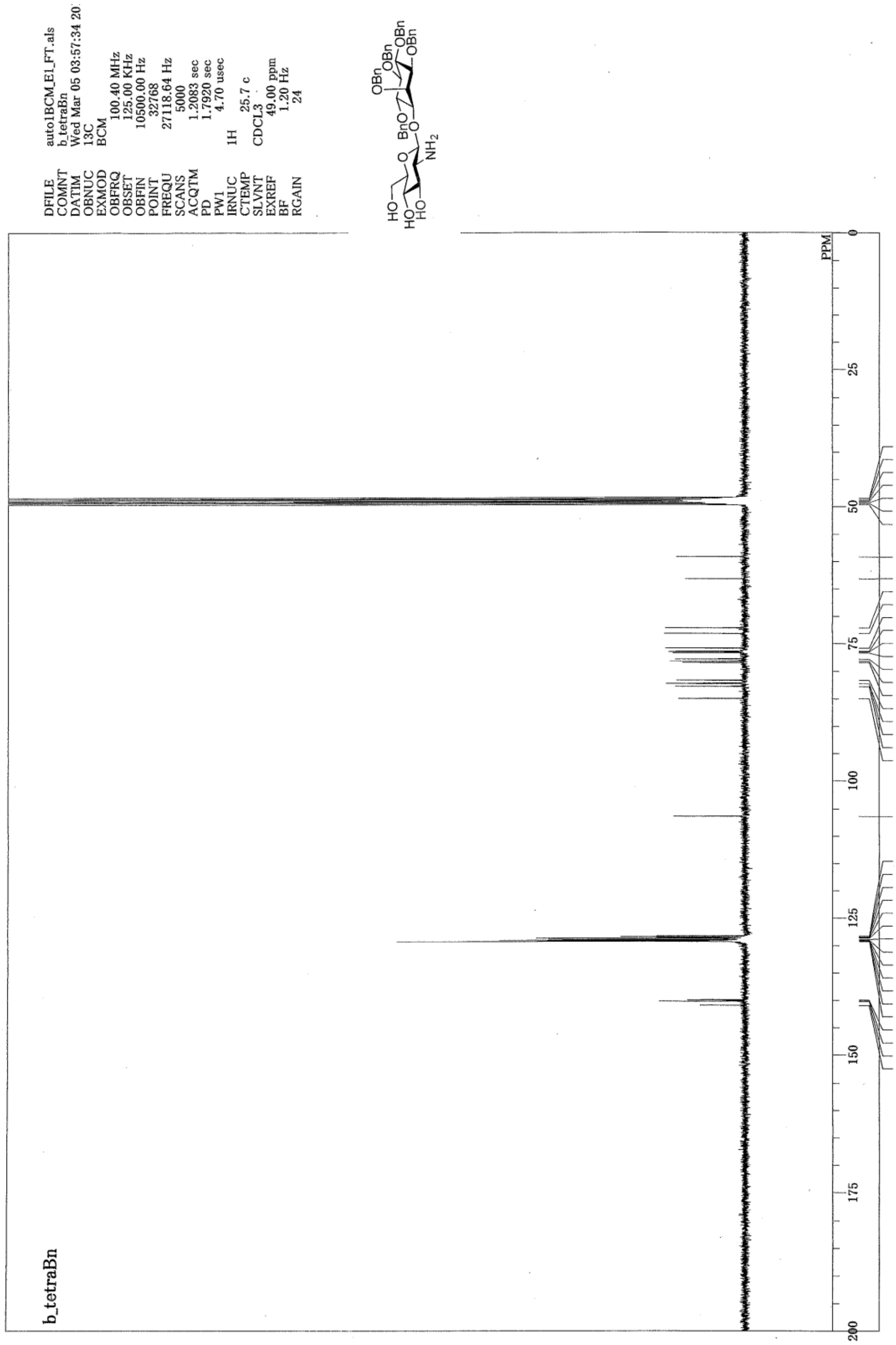

^{1}H NMR spectrum of **7**

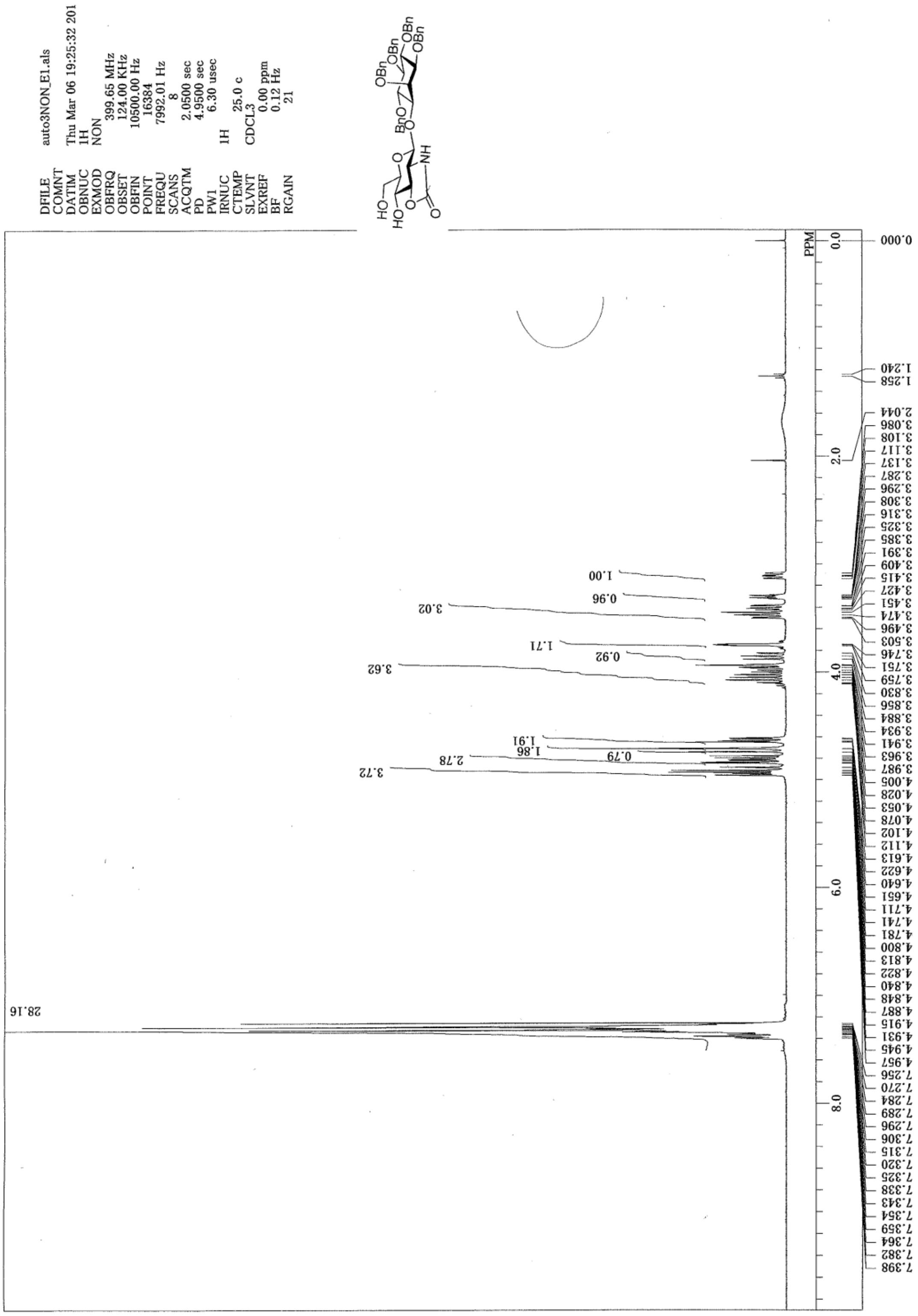

^{13}C NMR spectrum of **7**

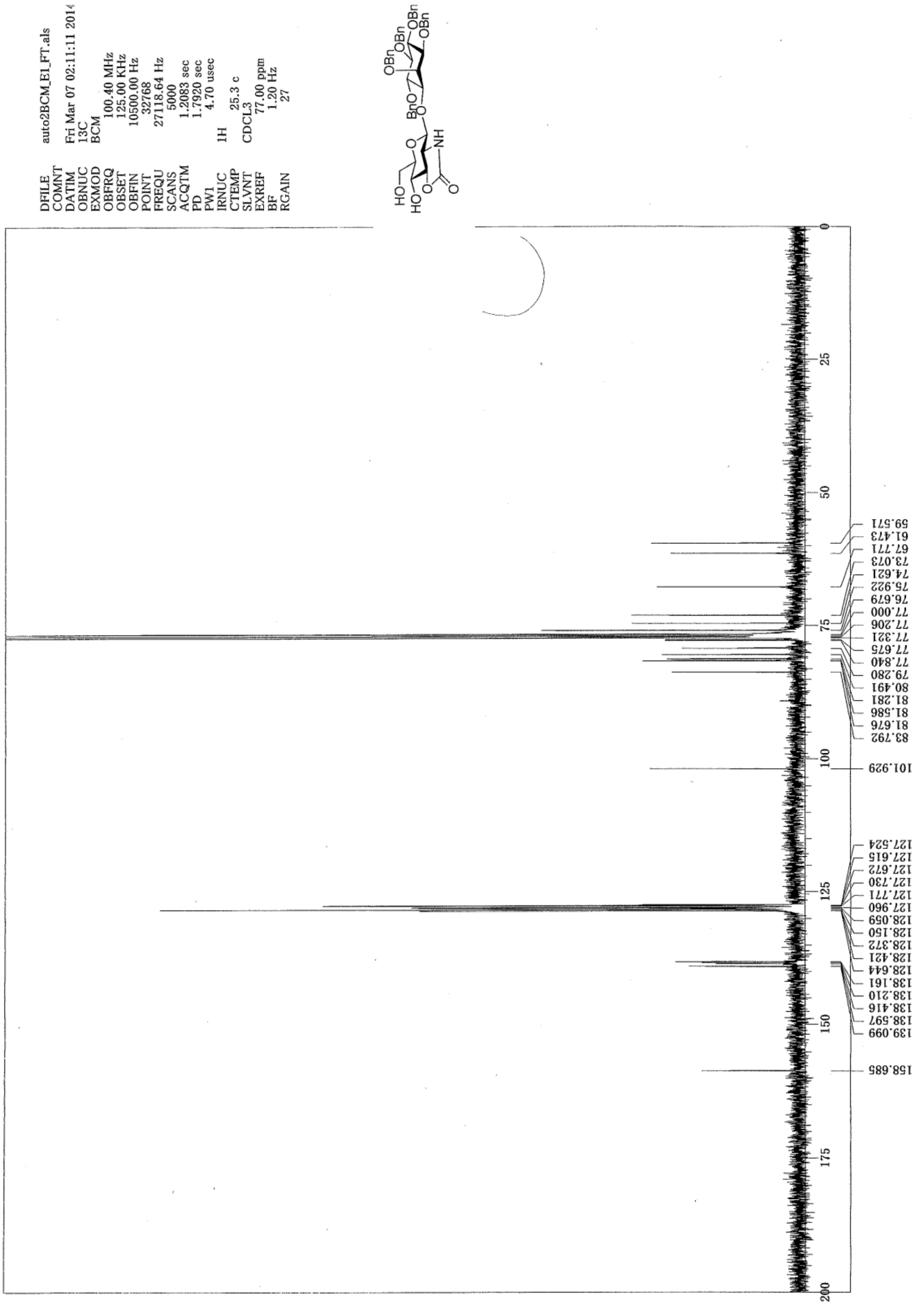

^{1}H NMR spectrum of **3**

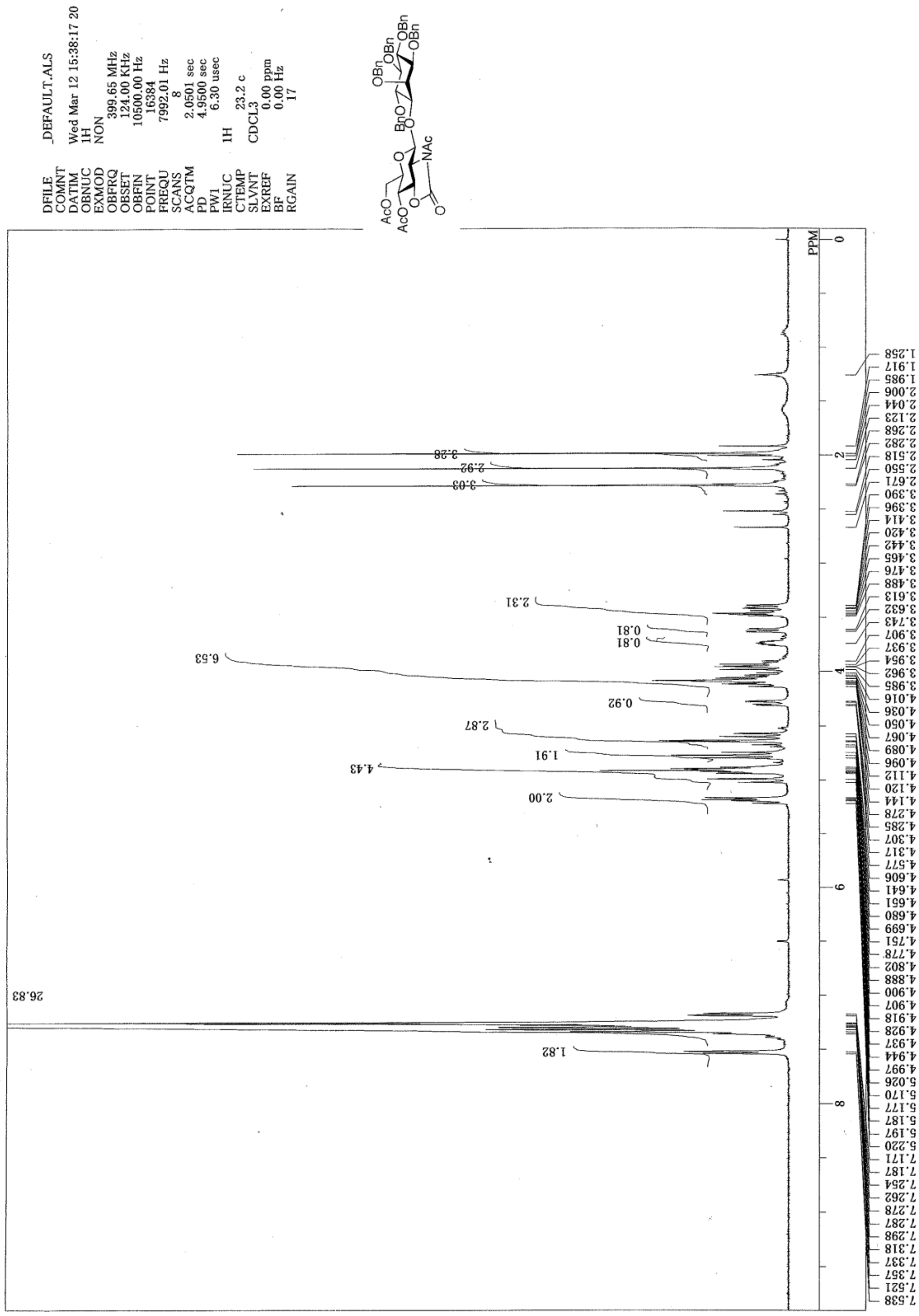

^{13}C NMR spectrum of **3**

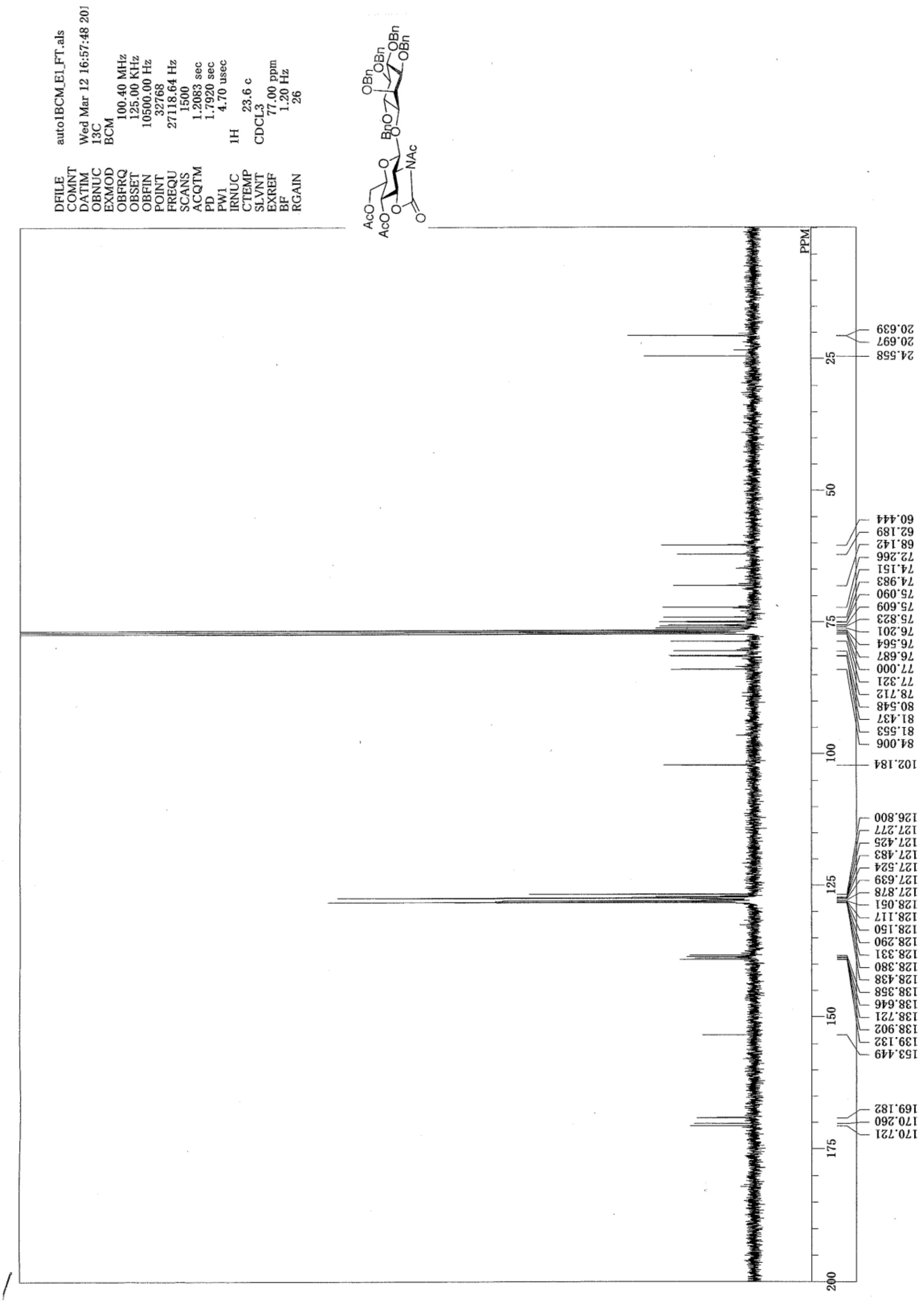

^{1}H NMR spectrum of **4**

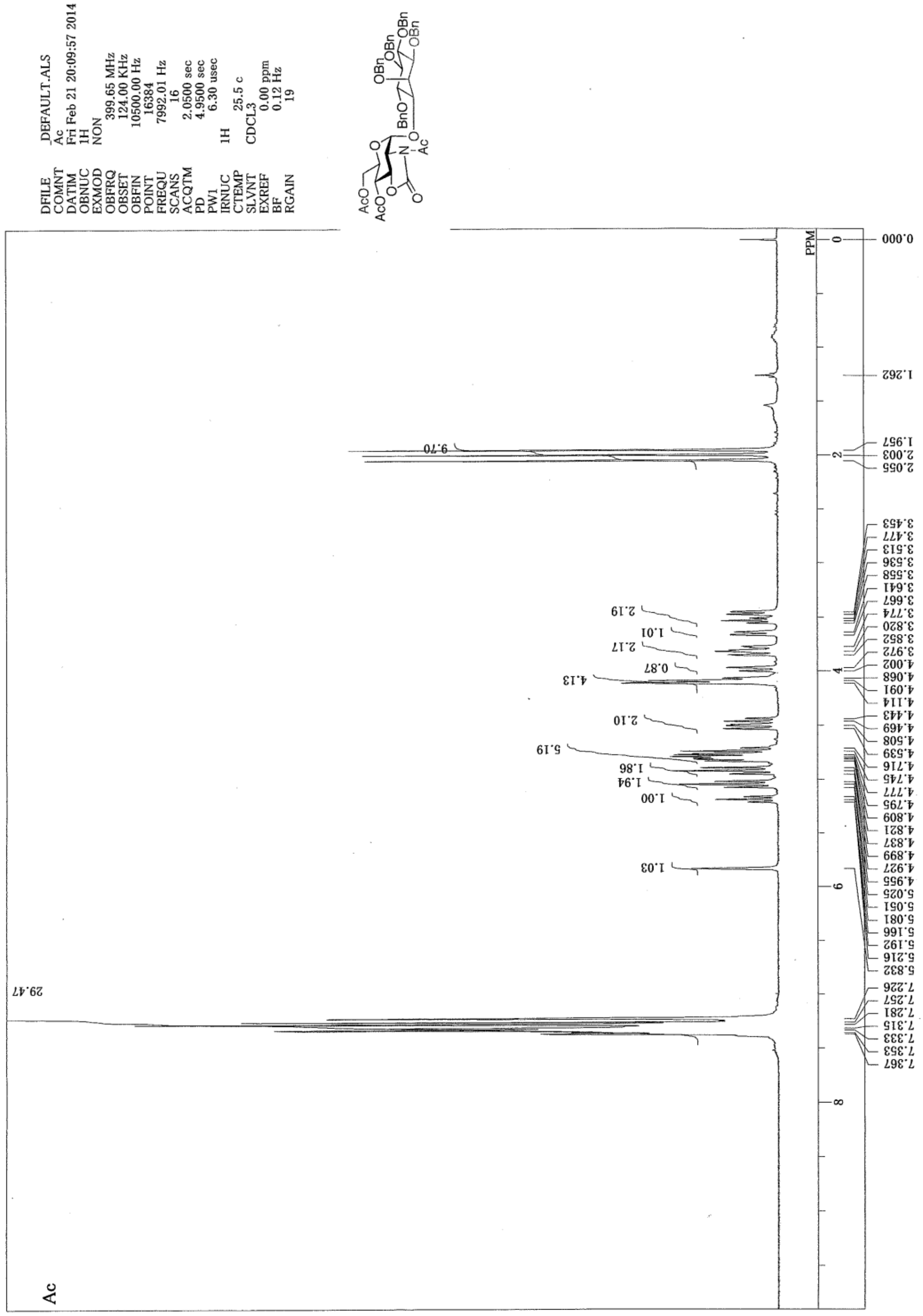

^{13}C NMR spectrum of **4**

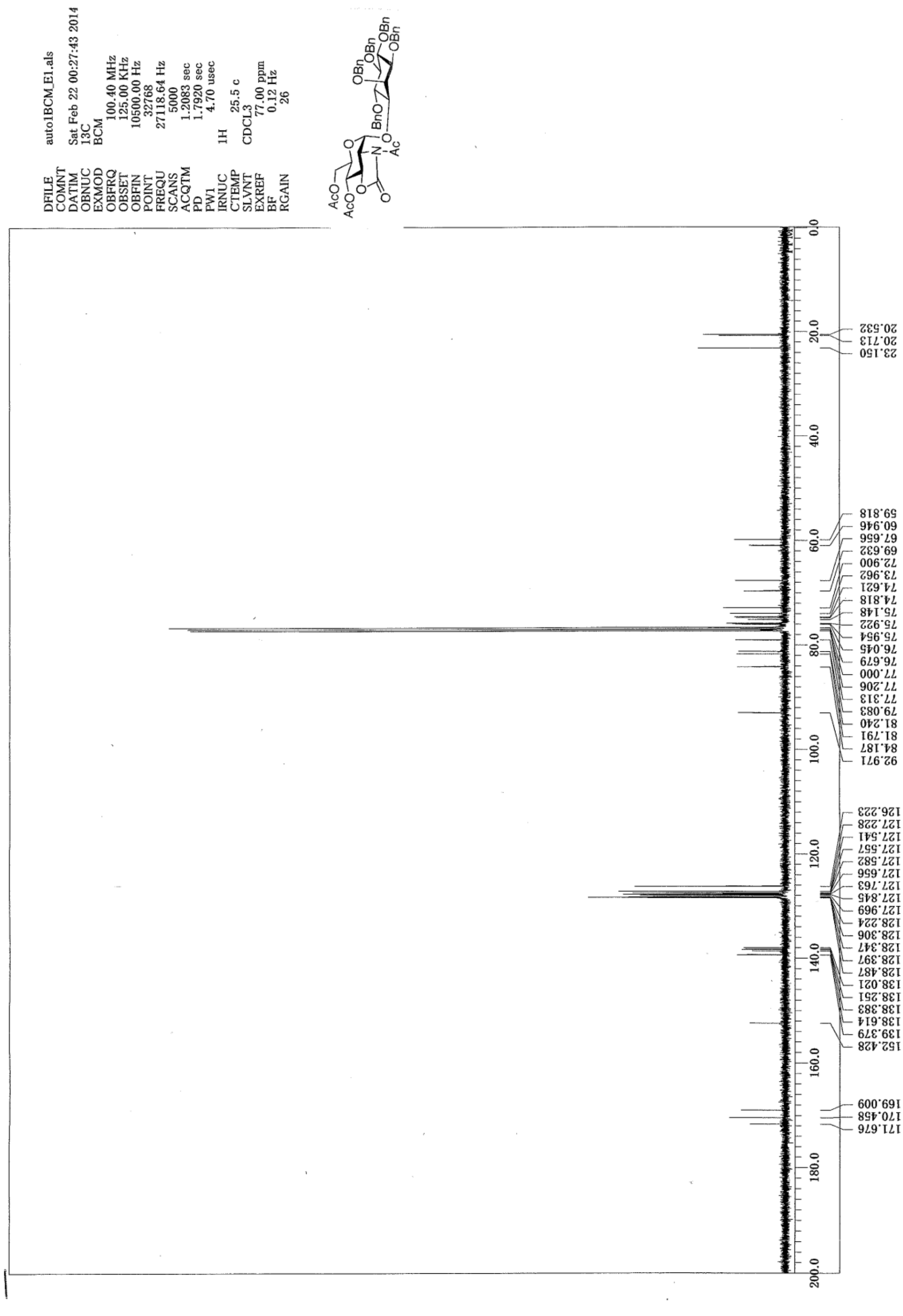

^{1}H NMR spectrum of **8**

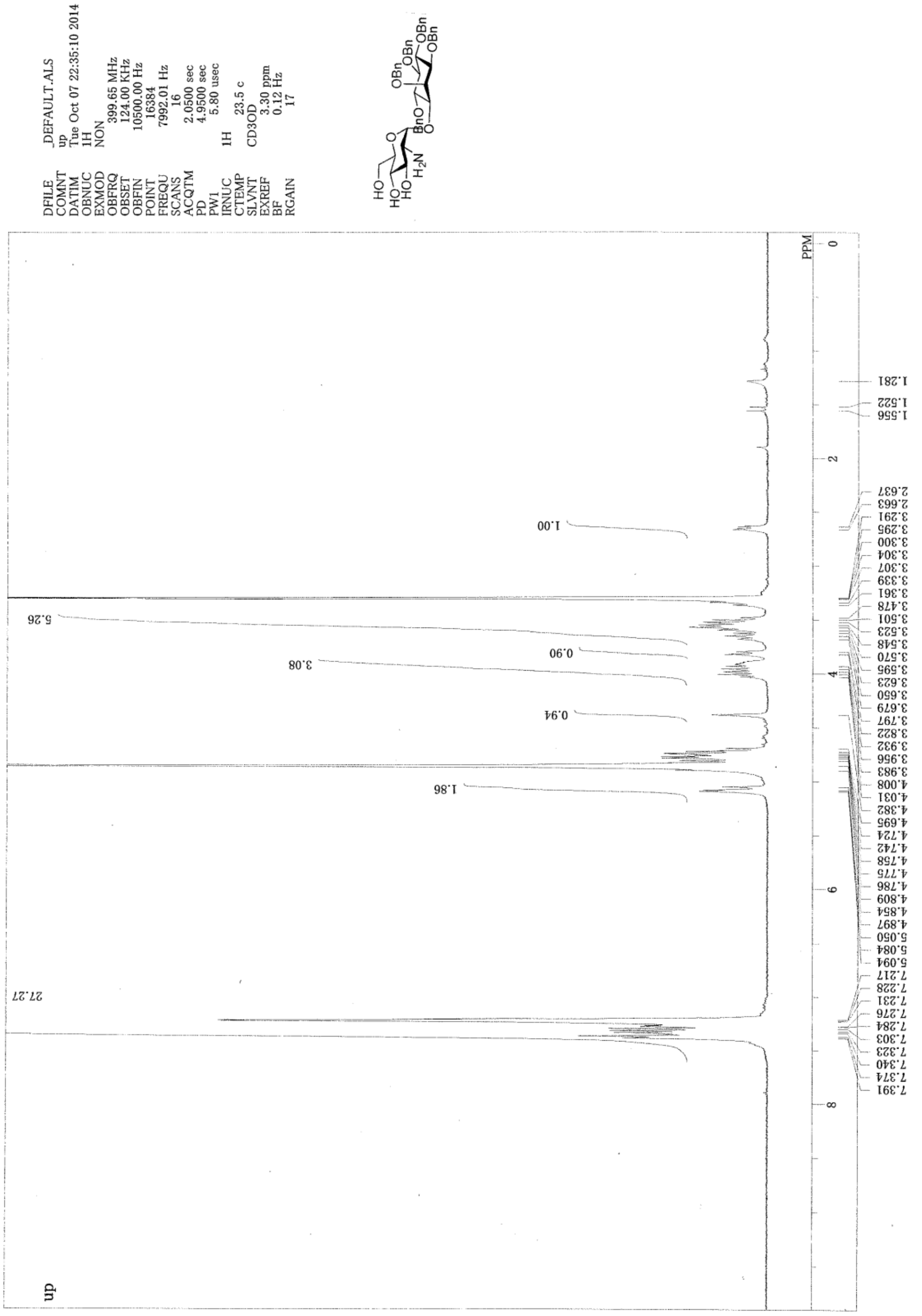

^{13}C NMR spectrum of **8**

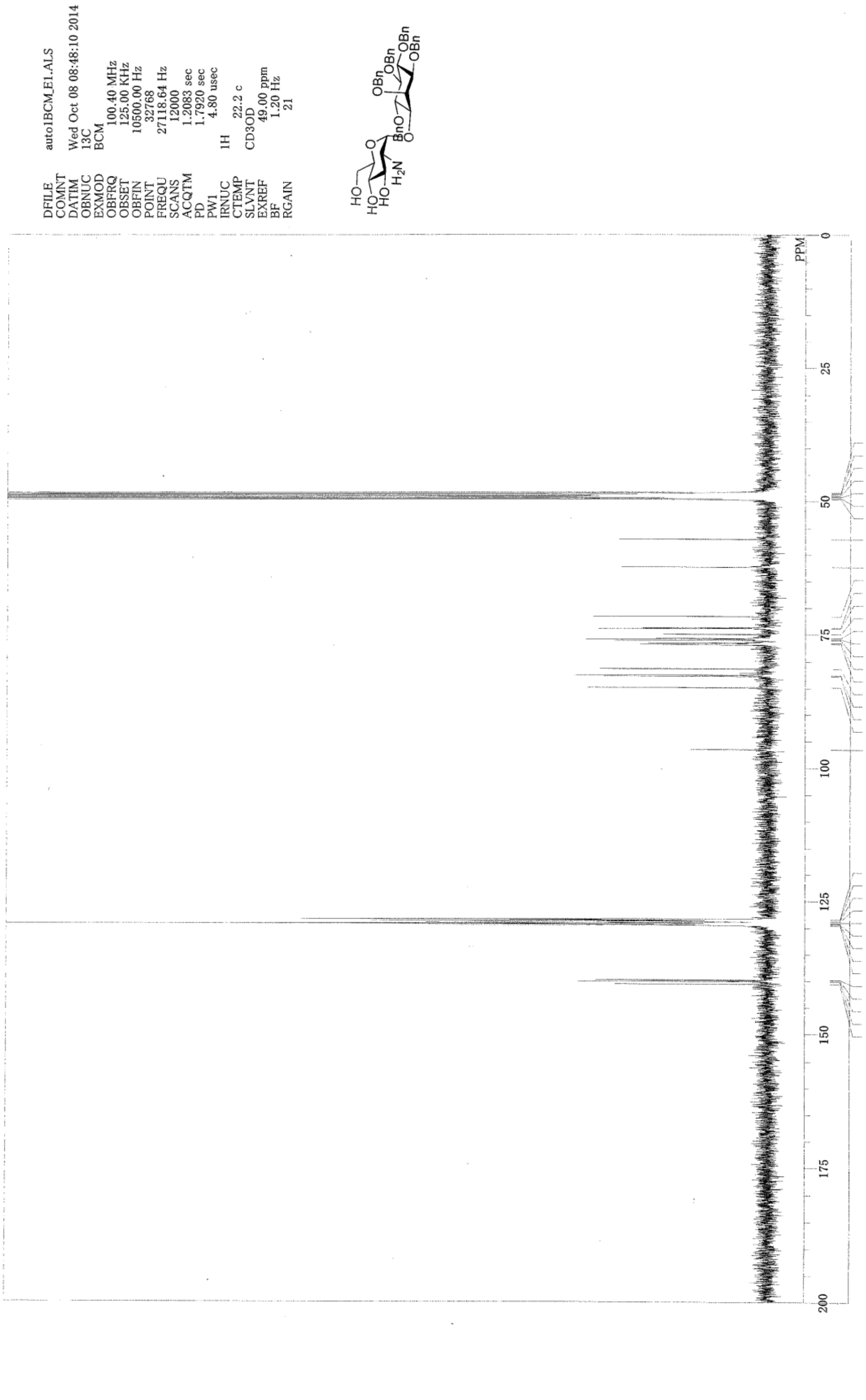

^{1}H NMR spectrum of debenzylated **8**

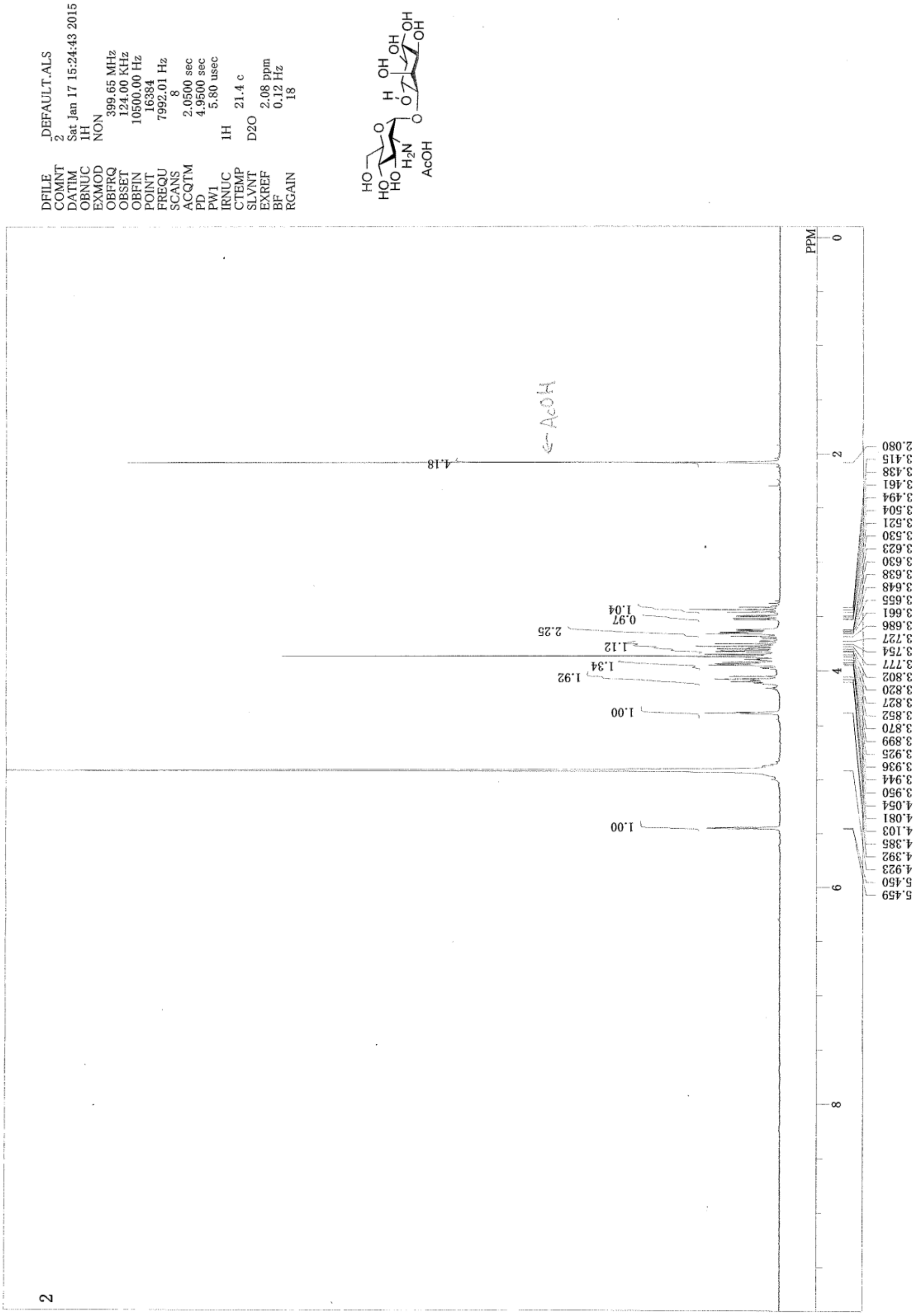

^{13}C NMR spectrum of debenzylated **8**

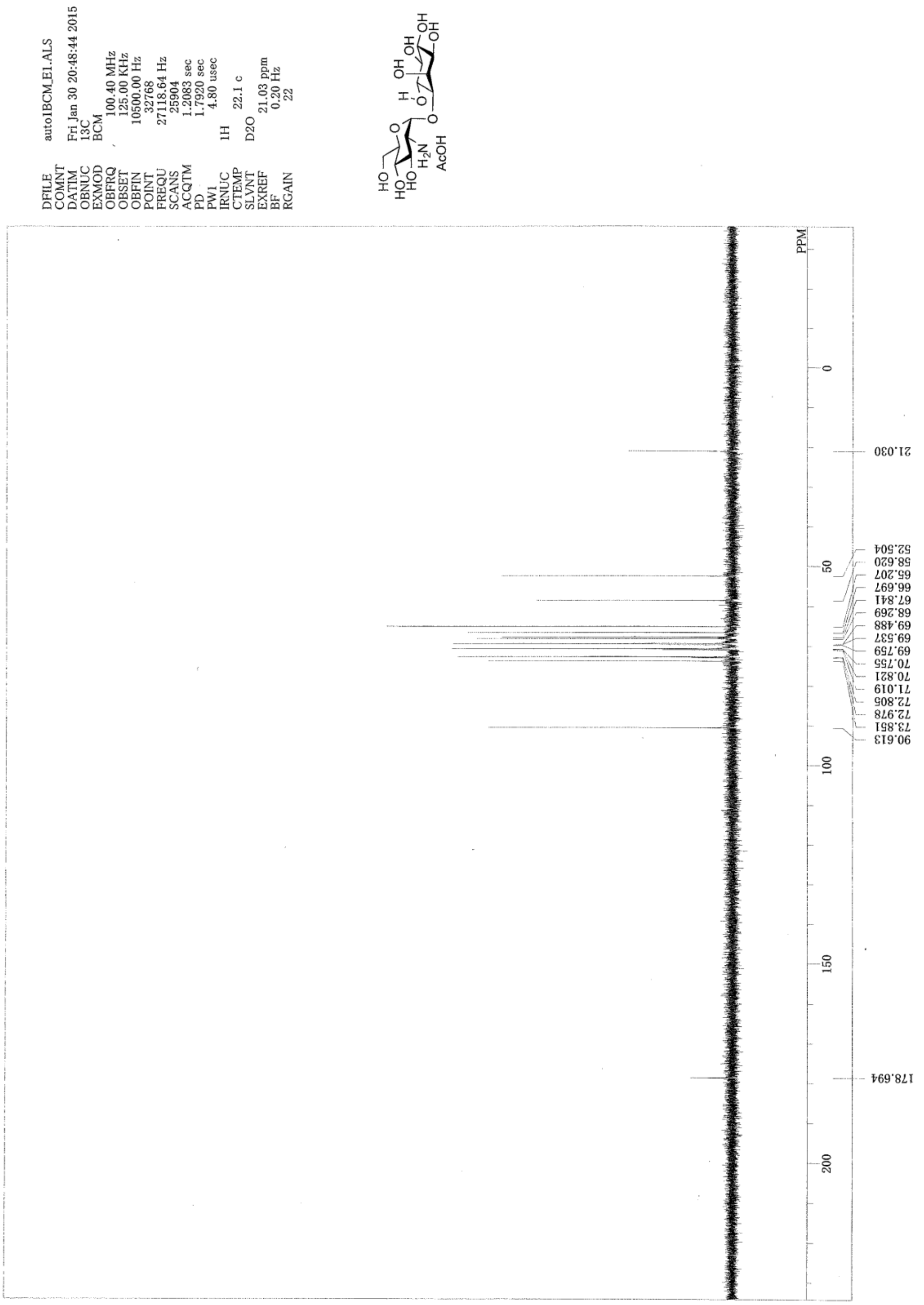

^{1}H NMR spectrum of **9**

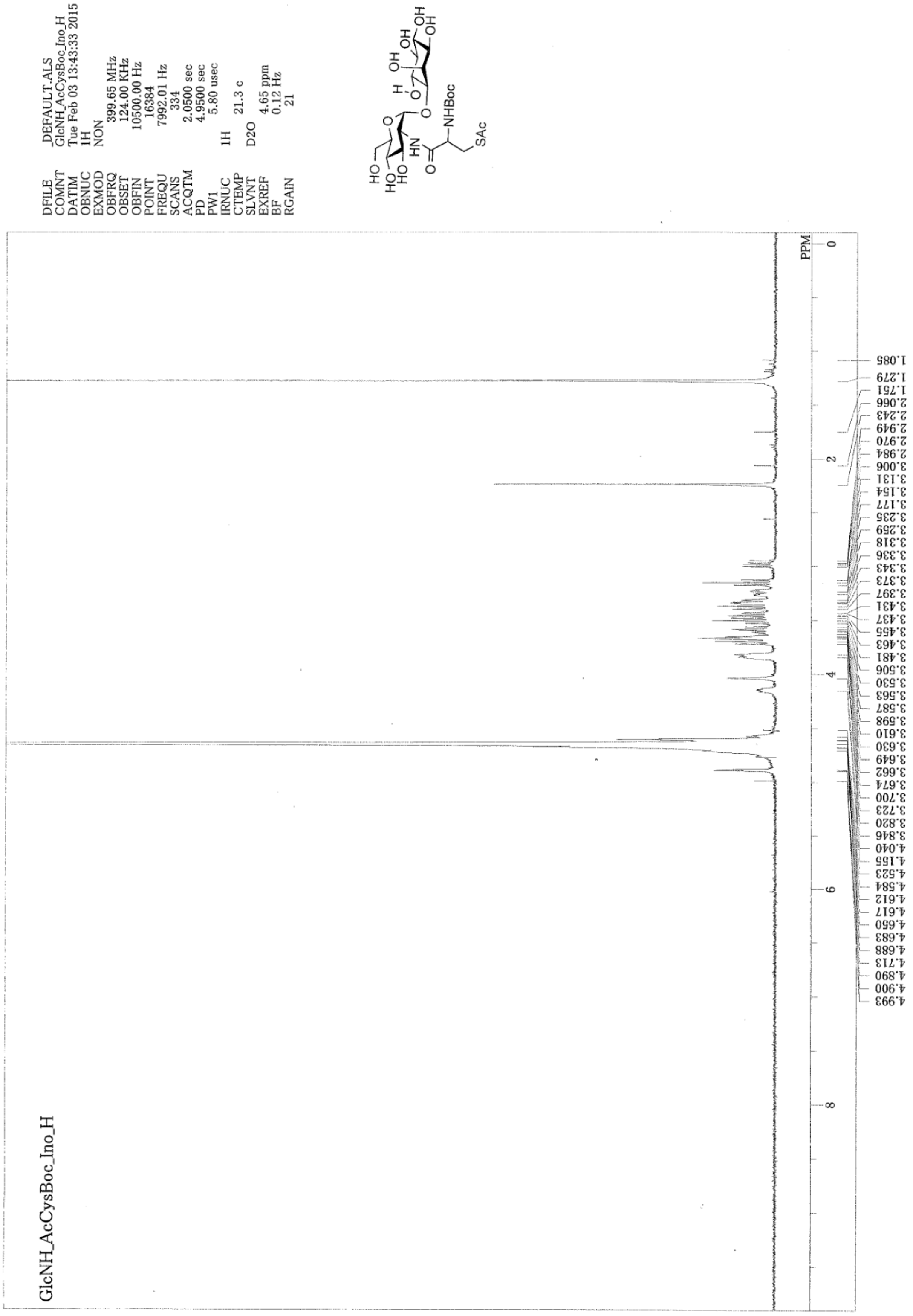

^{13}C NMR spectrum of **9**

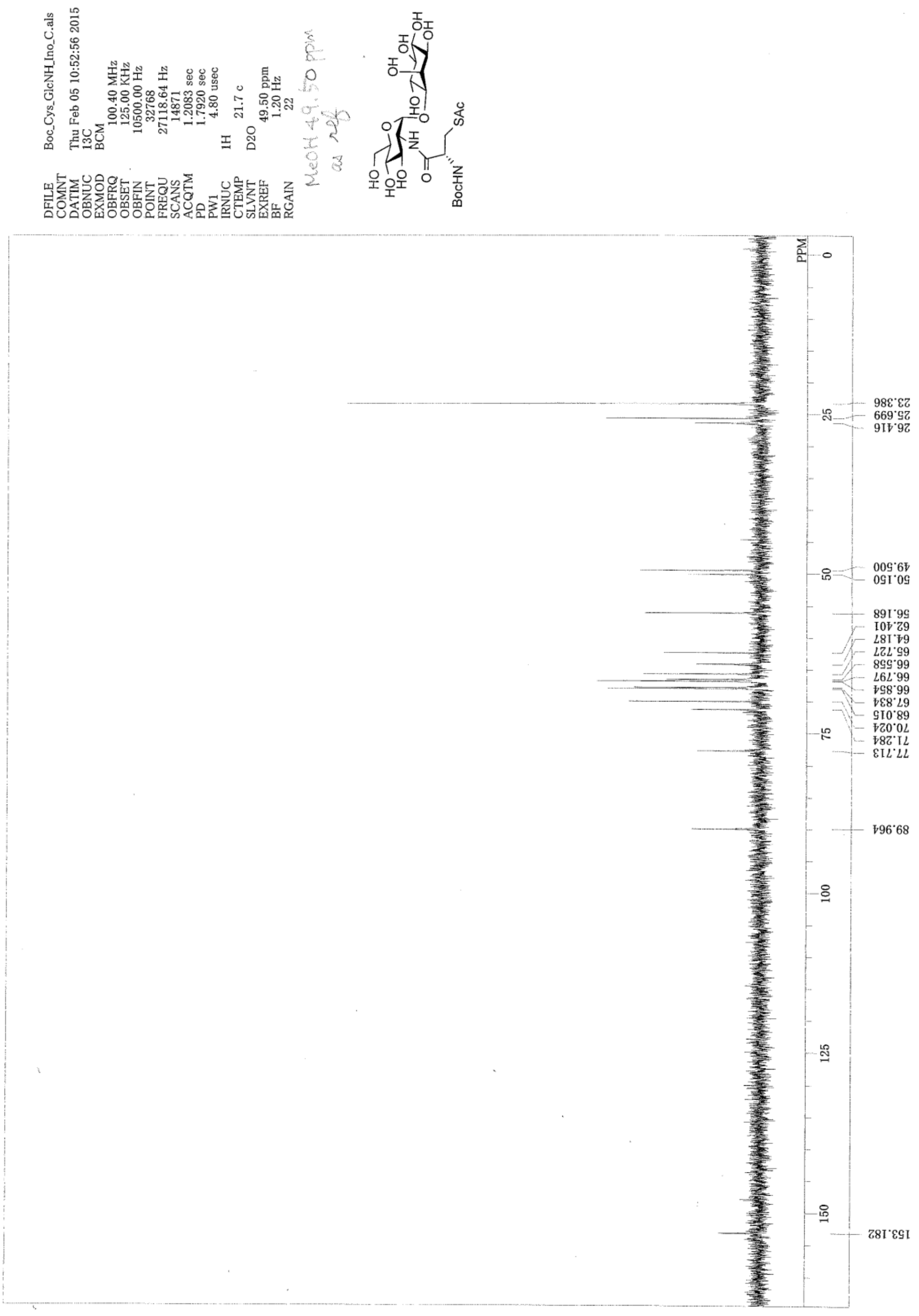

^{1}H NMR spectrum of mycothiol **1**

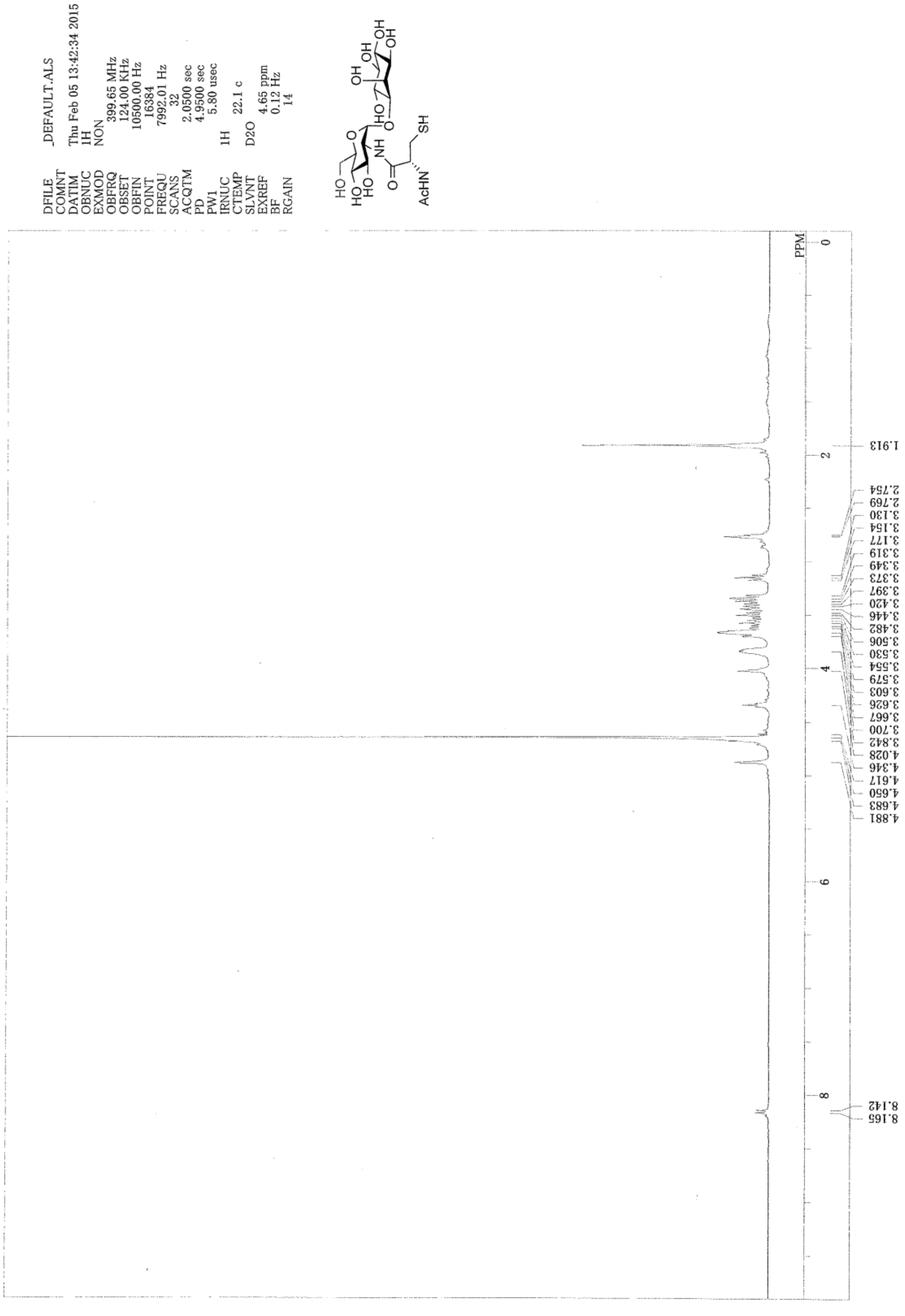

^{13}C NMR spectrum of mycothiol **1**

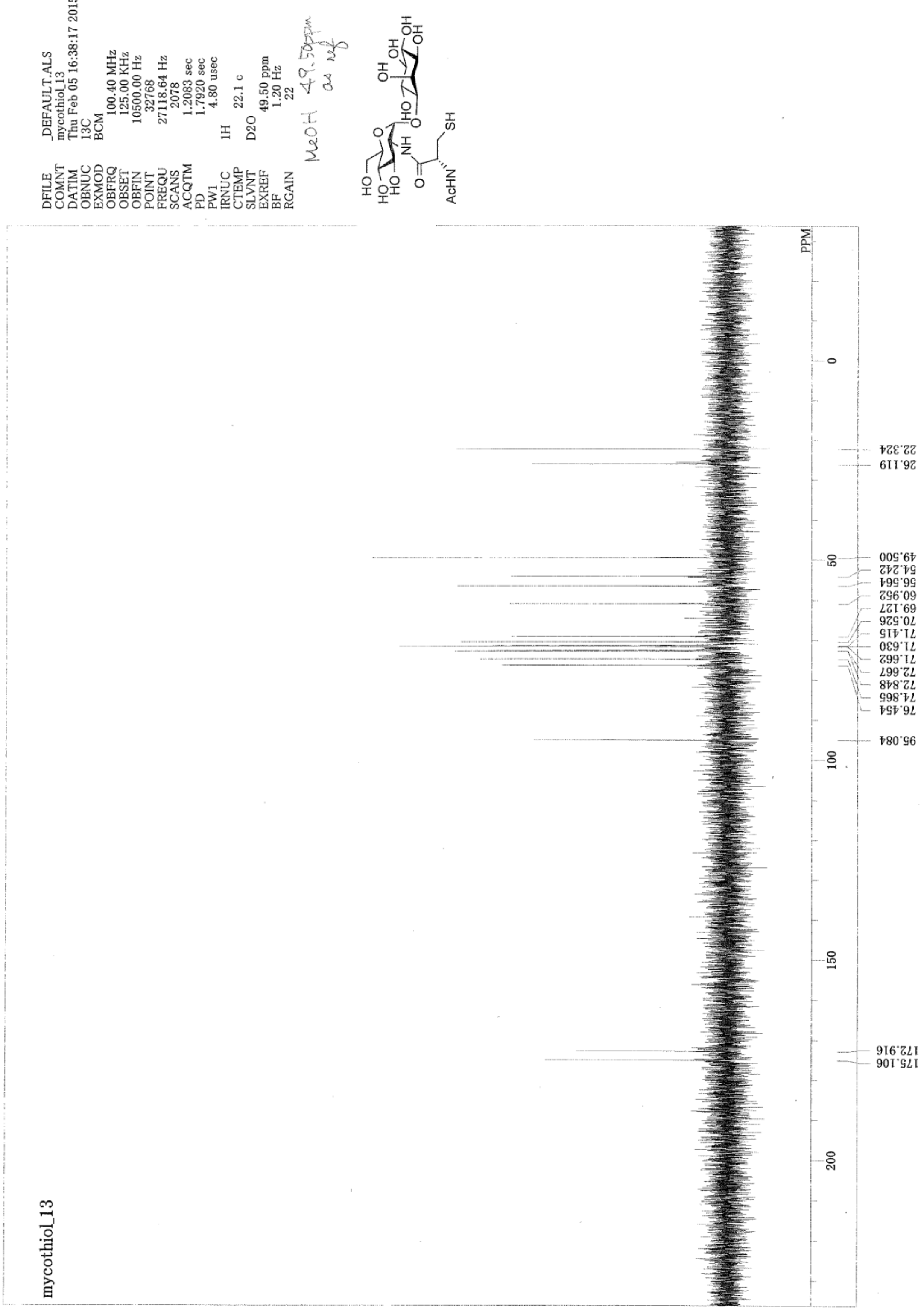

Supplement: File 1 — Experimental procedures, spectral data of new compounds, including 1H and 13C NMR spectra. [file Beilstein_J_Org_Chem-12-328-s001.pdf]
